# Risk factors for low birth weight among rural and urban Malaysian women

**DOI:** 10.1186/s12889-019-6864-4

**Published:** 2019-06-13

**Authors:** Satvinder Kaur, Choon Ming Ng, Slyvia E. Badon, Rohana Abdul Jalil, Dhivyalosini Maykanathan, Hip Seng Yim, Hamid Jan Jan Mohamed

**Affiliations:** 1 Faculty of Applied Sciences, UCSI University, Kuala Lumpur, Malaysia; 2 Division of Research, Kaiser Permanente Northern California, Oakland, California USA; 3 School of Medical Sciences, Universiti Sains Malaysia, Kubang Kerian, Kelantan Malaysia; 4 School of Health Sciences, Universiti Sains Malaysia, Kubang Kerian, Kelantan Malaysia

**Keywords:** Physical activity, Low birth weight, Rural, Urban, Malaysia

## Abstract

**Background:**

Low birth weight prevalence in Malaysia remains high. Socioeconomic background may lead to differences in physical activity and maternal nutritional status, which may play an important role in birth outcomes.

**Methods:**

This prospective cross-sectional study aimed to identify rural-urban differences in risk factors for low birth weight among women in Malaysia. Pregnant women at ≥20 weeks of gestation in urban and rural Malaysia (*n* = 437) completed questionnaires on sociodemographic characteristics and physical activity. Weight and middle-upper arm circumference were measured. Infant birth outcomes were extracted from medical records.

**Results:**

The overall prevalence of low birth weight infants was 6.38%. Rural women had more low birth weight infants than urban women (9.8% vs 2.0%, *p* = 0.03). Findings showed rural women were less sedentary (*p* = 0.003) and participated in more household/caregiving activities (*p* = 0.036), sports activities (*p* = 0.01) and less occupational activity (*p* < 0.001) than urban women. Logistic regression revealed that older age (OR = 1.395, 95% Cl = 1.053 to 1.846), low parity (OR = 0.256, 95% Cl = 0.088–0.747) and low middle-upper arm circumference (OR = 0.738, 95% Cl = 0.552 to 0.987) increased the risk of low birth weight infants in rural, but not in urban women.

**Conclusions:**

We observed differences in risk factors for low birth weight between urban and rural pregnant women. Age, malnutrition and low parity were risk factors for low birth weight among rural pregnant women. Our findings suggest that rural pregnant women with low nutritional status should be encouraged to monitor their middle-upper arm circumference consistently throughout pregnancy. Improving nutritional status in rural pregnant women may reduce the risk of low birth weight infants in this population.

## Background

Women from developing countries especially from Asia are more prone to give birth to infants with low birth weight (LBW) as compared to women from developed countries [1]. Based on the National Health and Morbidity Survey (NHMS) in Malaysia, 9.7% of children younger than 5 years old were born with LBW [2]. Infant birth weight has a strong role in determining short- and long-term health, thus reducing the prevalence of LBW should be given much importance. There are many risk factors for LBW including poor maternal nutrition and lifestyle factors (alcohol, tobacco use, drug abuse), pregnancy complications such as hypertension, low socio-economic conditions, maternal age, maternal body composition and parity [1, 3]. Maternal health and nutritional status are modifiable risk factors, that are particularly crucial in determining infant birth weight [1]. Poor maternal nutrition before and during pregnancy is a known cause of LBW in many developing countries [4]. The intake of sufficient energy, protein and nutrients to meet maternal and fetal requirements is required for optimal growth and birth weight [5]. However, the influence of maternal nutrition on LBW may be modified by environmental factors [5].

In urban and rural areas in Malaysia, the prevalence of LBW was 9.5 and 10.0% respectively [2]. The urban and rural areas differ not only in their geographical and environmental context, but also in the characteristics of residents and their household [6]. Many studies in developing countries have compared lifestyle in urban and rural areas and showed major disadvantages in rural areas. Rural women have lack of access to antenatal care [7], lower nutrition awareness [8] and poor diet quality [9] compared to urban women.

It has been shown that rural women have a higher risk for LBW than urban women [10]. However, advancement in technology especially in the field of mass media has crept into the lives of rural households mimicking the urbanites. Life in rural area is no longer traditional, with practices and habits in rural areas similar to the urban population [11]. Due to these changes, generalizing the effect of urban and rural settings on maternal and infant characteristics could be misleading, particularly when such factors have not been explored in Malaysia.

Apart from maternal nutritional status, lifestyle factors such as physical activity are important during pregnancy. Recommendations for regular physical activity during pregnancy have been advocated until late pregnancy, provided there are no other complications that arise during gestation period. Countries worldwide recommend moderate intensity aerobic physical activity ranging from 15 to 30 min at least 2 days up to 7 days a week [12]. Moderate physical activity is beneficial during pregnancy in prevention of obesity-related complications, stillbirth, and improving fitness and mode of giving birth [13].

Based on a study conducted in 3 states in Malaysia, almost 61% of adults were physically inactive with women being more inactive than men [14]. Parallel with rapid urbanization and modernization in Malaysia, women are employed in the professional field which may lead to low level of physical activity [15, 16]. However, there are still a number of women residing in rural settings that are involved in traditional roles which include household chores and agricultural activities [17]. This could cause a markedly different level and type of physical activity between both groups. As more Malaysian women become physically inactive, exploring the physical activity level and type among pregnant women from different demographic background could help to understand the differences in the activities conducted between the groups and whether these differences are associated with the outcome of LBW.

To our knowledge, there are limited studies that explored the risk factors for LBW among Malaysian women by comparing urban and rural differences. Most local studies in Malaysia focused on the urban areas or did not stratified according to residential areas which lead to findings being diluted by urban women [18, 19]. Understanding rural and urban differences in risk factors for LBW could govern proper intervention for targeted populations to improve the health of pregnant women with different demographic and lifestyle backgrounds in Malaysia. Identifying maternal factors leading to LBW in urban and rural Malaysian pregnant women would address the United Nations Millennium (2015) development goal to improve maternal health in low- and middle-income countries. In this study, there were 2 main objectives: (1) To determine differences in maternal physical activity and maternal characteristics of urban and rural pregnant women and (2) To identify risk factors for LBW among urban and rural pregnant women.

## Methods

### Study design and study population

We conducted a prospective cross-sectional study from February 2016 to January 2017, whereby pregnant women completed questionnaires during pregnancy and followed up after they had given birth. Malaysian pregnant women at ≥20 weeks of gestation, aged 19–40 that were willing to participate with informed consent form were included in the study. Women were recruited from urban and rural government antenatal clinics in Selangor, Malaysia. Selangor was chosen to better represent urban and rural vicinity which is the only state in Malaysia undergoing rapid urbanization and development with intense agricultural activities that represented the rural areas [20]. By using stratified random sampling, forty-five antenatal government clinics were selected from a list of 152 clinics (42 urban; 110 rural). In total, 13 urban and 32 rural clinics were visited for participant recruitment. Convenience sampling was used to recruit pregnant women. Our target sample size was 452 women, based on 80% power to detect a 14.3% prevalence of LBW [2]. After excluding women with multiple pregnancies, disabilities, or who were unable to communicate well in English or Malay language, 498 pregnant women were recruited. All participants provided their informed consent to participate in the study prior to data collection.

### Data collection

At study visit, participants completed questionnaires on sociodemographic characteristics and physical activity. This was followed with measurement of current weight and MUAC by trained staffs. At follow up, participants were contacted through phone call one week after their due date of birth to record birth information. Infants anthropometric outcome data at birth were obtained from medical records.

### Measures and outcomes

#### Sociodemographic background

Sociodemographic information was assessed using a questionnaire. It consisted of questions on maternal age, ethnicity, marital status, education level, occupation and monthly household income of the participants.

#### Pregnancy physical activity

Physical activity was measured using the Pregnancy Physical Activity Questionnaire (PPAQ) [21]. This self-administered questionnaire comprises of 32 close-ended questions on the average duration spent for household/caregiving, occupational, sports/exercise, transportation and sedentary activities, with 2 open-ended additional activities. Questions were scored according to the PPAQ scoring protocol [21]. Compendium-based metabolic equivalent (MET) values of every activity were used to estimate intensity [22]. Activities were further classified by intensity level after obtaining average weekly energy expenditure (METSs-h/week): sedentary (< 1.5 METs), light (1.5- < 3.0 METs), moderate (3.0–6.0 METs) and vigorous (> 6.0 METs) as well as type of physical activity (household, occupation, sport).

#### Maternal characteristics

Parity (number of previous births) was self-reported. Both pre-pregnancy weight and height was self-reported by pregnant women at study visit, which derived pre-pregnancy Body Mass Index (BMI). Pre-pregnancy BMI was categorized into four groups (underweight, normal weight, overweight and obese) based on the World Health Organization (WHO) classification of weight status [23]. A scale (Tanita SC-330) was used to measure weight at the study visit. Rate of gestational weight gain was calculated by subtracting pre-pregnancy weight from pregnancy weight at study visit, then dividing it by gestational week at study visit. Using IOM guidelines, rate of weight gain was categorized as inadequate, normal or excessive [24]. In addition, MUAC was measured using a non-stretchable measuring tape, midway between the olecranon of elbow and the acromion process of shoulder of the non-dominant arm.

#### Infant birth outcomes

Infant weight, length and head circumference at birth were obtained from medical records. WHO guidelines were used to categorize infant birth weight into low birth weight (< 2.5 kg), normal (2.5–4.0 kg) and high birth weight (> 4.0 kg) [25]. Birth week and mode of birth (vaginal or assisted birth) were self-reported through follow-up phone call.

### Ethics approval

Approval to conduct the study in government maternal clinics was granted by National Medical Research Registrar, (NMRR)-15–1532-26,422 and Medical Research Ethics Committee (MREC) (KKM/NIHSEC/P15–1362) on the 18th November 2015. In addition, the directors of all Health District offices provided their consent for data collection prior to the assessment day.

### Statistical analysis

Results were presented as mean (standard deviation) for continuous variables and frequency (percentage) for categorical variables. Data that were not normally distributed were presented as median (25th and 75th percentiles). All descriptive statistics were presented separately in rural and urban women. An independent student t-test was used to test differences between urban and rural women for normally distributed variables. Mann-Whitney test and Kruskal-Wallis one-way ANOVA was used to test differences between urban and rural women for non-normally distributed data. Chi-square or Fisher’s Exact test was used to test differences between urban and rural pregnant women for categorical variables. Logistic regression was used to determine risk factors for LBW infants. Models were run separately for rural and urban pregnant women. Each maternal characteristic that was associated with LBW in univariable models (Pearson correlation *p* < 0.05) was included in a multivariable model. Variables included in the multivariable model were age, delivery week, parity and MUAC (cm). All analyses were done with IBM SPSS Statistics (Version 22.0, SPSS, Chicago, IL). Statistical significance was set at *p* < 0.05.

## Results

### Participants

A total of 498 pregnant women were consented for the study. However, 61 of them were excluded due to incomplete data. At follow-up, 54% (*n* = 235) of participants responded to phone interviews. Some women were lost to follow up [*n* = 202 (102 urban women, 133 rural women)] due to inactive phone numbers, difficulty finding time for the interview and dropout as they chose not to participate in the study. Sociodemographic characteristics of 437 participants are presented in Table 1. There were significant differences between urban and rural pregnant women in terms of parity (*p* = 0.02), ethnicity (p = 0.02), education level (*p* < 0.001), monthly household income (p < 0.001) and occupation (*p* < 0.001).

**Table 1:** Maternal characteristics in urban and rural area (*n* = 437)

Characteristics	Urban (*n* = 199)	Rural (*n* = 238)	*p*-value
Age (years)^a^	29.54 (4.76)	29.19 (5.23)	0.44
Gestational age at study visit, (weeks)^a^	30.46 (5.52)	30.19 (5.82)	0.62
Second Trimester (≥20–26 weeks) (%)	49 (24.62)	75 (31.51)	0.11
Third Trimester (≥27 weeks) (%)	150 (75.38)	163 (68.49)	
Parity (%)			
0	91 (45.7)	79 (33.2)	0.01*
1–2	57 (28.6)	70 (29.4)	
≥ 3	51 (25.6)	89 (37.4)	
Ethnicity (%)			
Malay	145 (72.9)	182 (76.5)	0.02*
Chinese	26 (13.1)	12 (5.0)	
Indian	28 (14.1)	44 (18.5)	
Marital Status (%)			
Married	195 (98)	237 (99.6)	0.06
Single/Widowed	4 (2)	1 (0.4)	
Education Level (%)			
Primary School	4 (2)	10 (4.2)	< 0.001*
Secondary School	70 (35.2)	133 (55.9)	
Tertiary School	125 (62.8)	95 (39.9)	
Monthly Household Income (%)^b^			
Low (< RM2500)	58 (29.1)	143 (60.1)	< 0.001*
Middle (RM2500-RM5599)	105 (52.8)	77 (32.4)	
High (≥ RM5600)	36 (18.1)	18 (7.6)	
Occupation (%)			
Managers & Professional	57 (28.6)	36 (15.1)	< 0.001*
Technician, Associate Professional, Clerical, Sales Workers	79 (39.7)	59 (24.8)	
Agricultural, Forestry, Fishery, Craft Workers, Plant and Machine Operators	6 (3)	10 (4.2)	
Unemployed	57 (28.6)	133 (55.9)	

^a^Reported in Mean (SD); *LMP* Last menstrual period, *RM* Ringgit Malaysia (1 USD = RM4.04);

^b^Based on the cut-off of 11th Malaysia Plan 2016–2020; * Significance value at *p* < 0.05

### Maternal physical activity level

Physical activity level of urban and rural pregnant women is shown in Table 2. Pregnant women in urban areas were more sedentary than rural pregnant women (*p* = 0.003). There was a significant difference between pregnant women in urban and rural areas for every domain of physical activity. Rural pregnant women were more active in household and sports physical activity (*p* = 0.036 and < 0.010, respectively), whereas urban pregnant women were more active in occupational physical activity (p < 0.001). Both urban (*p* = 0.001) and rural pregnant women (p = 0.001) with higher education background had higher physical activity level (Table 3). Birth mode and maternal age was not associated with physical activity level in both urban and rural women.

**Table 2:** Maternal physical activity level in urban and rural pregnant women (*n* = 437)

	Urban n = 199	Rural n = 238	
Intensity of Physical Activity (MET-hours/week)	Median (25th, 75th)	Median (25th, 75th)	*p*-value
Total	186.1 (130.0, 248.5)	184.8 (127.6, 247.3)	0.748
Sedentary	74.2 (41.7, 104.4)	58.0 (30.7, 96.0)	0.003*
Moderate	15.8 (0.8, 44.8)	15.8 (4.8, 47.9)	0.383
Light	79.5 (52.2, 108.8)	88.2 (57.6, 115.7)	0.111
Vigorous	0 (0, 0)	0 (0, 0)	0.228
Type of Physical Activity			
Household	76 (45.2, 113.9)	82.8 (52.5, 124.6)	0.036*
Occupational	49 (0, 86.5)	0 (0, 71.1)	< 0.001*
Sports	0 (0, 2.4)	0.38 (0, 3.5)	0.010*

*Significance value at *p* < 0.05 using Mann-Whitney U test

**Table 3:** Differences in factors of physical activity of urban and rural pregnant women (n = 437)

	Urban n = 199		Rural n = 238	
Intensity of Physical Activity (MET-hours/week)	Median (25th, 75th)	*p*-value	Median (25th, 75th)	*p*-value
Maternal age				
17–26	165.2 (121.6, 221.2)	0.064	184.7 (125.4, 242.5)	0.05
27–36	194.1 (136.1, 250.2)		187.1 (130.5, 269.9)	
37–46	237.1 (139.7, 294.4)		126.6 (95.0, 209.6)	
Education level				
Primary school	124.9 (58.2, 239.7)	0.001*	131.1 (109.6, 168.0)	0.001*
Secondary school	170.2 (120.7, 209.5)		171.5 (117.1, 218.7)	
Tertiary school	205.3 (154.3, 262.2)		224.4 (145.4, 293.5)	
Delivery Mode^a^				
Vaginal delivery	195.5 (135.9, 240.5)	0.594	176.8 (120.8, 257.1)	0.818
Assisted delivery	177.2 (137.2, 237.6)		175.4 (125.2, 227.4)	

*Significance value at *p* < 0.05 using Kruskal-Wallis One-Way ANOVA

^a^Delivery Mode was tested using Mann-Whitney U test

### Maternal and infant characteristics

All 437 women completed weight assessment at the study visit however, at follow up, only 235 women provided the birth and infant outcomes data. Maternal and infant characteristics for urban and rural pregnant women are shown in Table 4. Rural women were more likely to be overweight or obese than urban women (39.5% vs 30.7%). Both urban and rural women had normal MUAC, between the cutoff points of 23 to 31 cm [26, 27]. There was no significant difference in infant birth weight between women in urban and rural areas. However, there were significant differences in infant birth weight category (*p* = 0.03), and head circumference at birth (*p* = 0.008). More pregnant women in rural areas had infants with low birth weight (10% vs 2%) compared to pregnant women in urban areas. Urban pregnant women (3.9%, *n* = 4) had greater incidence of high birth weight (3.9% vs 1.5%) than rural pregnant women. Head circumference of infants from urban districts was also significantly larger than rural districts (34.46 cm vs 33.32 cm).

**Table 4:** Maternal and infant characteristics

Variables	Urban	Rural	*p*-value
Gestational age at birth, weeks (*n* = 203)	39.30 (1.72)	39.52 (2.32)	0.44
Pre-pregnancy BMI (kg/m^2^)	23.66 (4.89)	24.50 (5.37)	0.079
Pre-pregnancy BMI category, n (%), (*n* = 437)			
Underweight	26 (13.1)	37 (15.5)	0.129
Normal	112 (56.3)	107 (45)	
Overweight	38 (19.1)	60 (25.2)	
Obese	23 (11.6)	34 (14.3)	
MUAC (cm) (n = 437)	27.21 (3.95)	27.93 (4.36)	0.068
Rate of gestational weight gain/week (kg) Gestational weight gain category, (%) (*n* = 235)	0.34 (0.21)	0.31 (0.16)	0.107
Inadequate	27 (26.4)	41 (30.8)	0.260
Normal	26 (25.5)	42 (24.2)	
Excessive	49 (48)	50 (37.6)	
Infant birth weight (kg)	3.18 (0.42)	3.09 (0.43)	0.101
Infant birth weight category (*n* = 235)			
Low	2 (2.0)	13 (9.8)	0.03*
Normal	96 (94.1)	118 (88.7)	
High	4 (3.9)	2 (1.5)	
Head Circumference at birth (cm) (*n* = 235)	34.46 (3.65)	33.32 (1.88)	0.008*
Birth length (cm) (*n* = 235)	50.39 (3.06)	49.66 (2.87)	0.100

*Significance value at *p* < 0.05

### Risk factors for low birth weight infant in urban and rural pregnant women

Logistic regression included factors found to significantly influence birth weight in univariate analysis (Pearson correlation *p* < 0.05). Physical activity, pre-pregnancy BMI and gestational weight gain were not correlated with birth weight and therefore were excluded from the logistic regression models. In rural pregnant women, higher MUAC (OR = 0.735, 95% Cl = 0.567 to 0.952) and greater parity (OR = 0.267, 95% Cl = 0.097–0.737) were associated with lower risk of birth to LBW infants (Table 5). On the other hand, increased maternal age (OR = 1.332, 95% Cl = 1.039 to 1.708) was associated with higher risk of LBW infant. Delivery week was not associated with infant’s birth weight. These predictors explained 31.7% variation in the adjusted model. In contrast, these predictors were not significantly associated with LBW in urban pregnant women.

**Table 5:** Factors associated with infant low birth weight^a^ (*n* = 197)

	Urban^b^			Rural^c^		
Variables	OR	95% CI	*p*-value	OR	95% Cl	*p*-value
Maternal age	1.311	0.824–2.087	0.253	1.332	(1.039–1.708)	0.024*
Parity	–	–	–	0.267	(0.097–0.737)	0.011*
MUAC	1.045	0.525–2.083	0.899	0.735	(0.567–0.952)	0.020*

^a^Regression was adjusted for factors found to significantly influence birth weight (in Pearson correlation *p* < 0.05). Delivery week was included in regression models; however, no significant difference was found. *Significant at *p* < 0.05

^b^Nagelkerke R^2^ = 0.317; χ^2^ = 19.4, p = 0.001 with df = 4

^c^Nagelkerke R^2^ = 0.360; χ^2^ = 5.458, *p* = 0.243 with df = 4

## Discussion

This was a comparative study of physical activity, maternal characteristics and risk factors for LBW among urban and rural pregnant women in Selangor, Malaysia. The ethnicities of pregnant women recruited were similar with the main ethnic composition of Malaysia, which includes the Malays, Chinese, Indians and other ethnicities. In the present study, there was a clear difference in ethnicity, parity, education level, monthly household income and occupation between pregnant women from urban and rural counterparts in Selangor. We also demonstrated that pregnant women from urban areas had higher level of education compared to pregnant women in rural areas, consistent with past studies in developing countries [8]. The higher level of education among urban pregnant women in this study was reflected in higher household incomes through securing occupations in a professional field whereby urban woman were employed in professional and administrative roles while rural women were more involved in domestic activities.

One of the objective of this study was to compare the level and type physical activity among urban and rural pregnant women. This was among the first studies to use PPAQ as a tool to assess differences in physical activity level between rural and urban pregnant women in Malaysia. Our findings showed that urban and rural pregnant women with higher education levels were more physically active than pregnant women with lower education levels, as shown earlier [13, 28]. Pregnant women with higher education levels may have more access to knowledge about physical activity and thus are more likely to exercise [29]. The higher sedentary activity of urban pregnant women as compared to their rural counterparts that we observed in this study was consistent with a recent study in China, which reported that urban pregnant women spend 34–40% of total energy expenditure in sedentary activities [28]. Socio-demographic data showed that urban women were more engaged in occupational activity with 66.5% of urban women in this current study working in a professional field or technician job, which may explain the sedentary activity. Tiredness, discomfort and insufficient time were factors contributing to sedentary activity among urban pregnant women in previous studies [28].

In this current study, rural pregnant women were more active in sports activities, such as walking and jogging, in their leisure time compared to urban women. However, median score for sports-related activity was lower among the Malaysians as compared to Turkish [30]. Most Malaysians appeared to devalue the importance or desirability of physical activity as a leisure-time pursuit [14, 31]. Findings from NHMS 2015 demonstrated that Malaysian adult women had a higher participation in light and moderate intensity activities, such as household chores as compared to vigorous intensity activities such as sports and exercise [16]. Nonetheless, Malaysian pregnant women still scored much lower as compared to the Turkish in light, moderate-intensity and household physical activity [30]. Although Malaysia and Turkey are both upper middle-income countries with similar economic standing, it seemed that Malaysian pregnant women do not engage in physical activity as much as the Turkish. Further research is needed to understand the behavioural and cultural influences on the lack of physical activity, barriers faced and physical activity taboos among Malaysian pregnant women.

Physical activity level of urban and rural women in our study was below recommendations, despite general guidelines for physical activity among Malaysian pregnant women are made available [32]. Thus, intervention programmes to promote physical activity among Malaysian pregnant women are crucial and should be tailored based on geographical area due to the distinct differences in lifestyle and the types of activity performed. For instance, physical activity interventions for urban pregnant women may include activities during office hours meanwhile household physical activity can be planned for rural pregnant women instead.

We then proceeded to identify risk factors for LBW in rural and urban women. Physical activity was not associated with risk of LBW infants both in rural and urban pregnant women, consistent with earlier studies [13, 33]. In a previous study, sports and vigorous activity during the first trimester of pregnancy were associated with increased risk for LBW infants, but no associations were found during the second and third trimesters of pregnancy [33]. Pregnant women in our study were in their second or third trimester of pregnancy and more than half did not engage in vigorous or sports-related activity, which may explain the lack of association between sports activity and LBW infants in our study.

No significant associations were found between maternal characteristics and LBW among urban pregnant women. Additionally, we demonstrated that more rural women had infants with LBW compared to urban women. It is well-established that adverse pregnancy outcomes are more prevalent among pregnant women with low socioeconomic status [5]. Based on socio-demographic data in this current study, rural women had lower educational attainment and income compared to urban women. Low socioeconomic status leads to restricted access to information and health services, prenatal care, material conditions which are closely tied to diet quality and nutritional status of rural women, fetal growth and thus increase the risk of adverse pregnancy outcomes such as LBW [5, 6].

Low MUAC, low parity and increased maternal age were associated with the risk of delivering LBW infants in rural pregnant women in this study. Pregnant women aged ≥40 years were at a higher risk for LBW than pregnant women aged 25–29 years [34]. However, a previous case-control study in Malaysia showed that younger maternal age was associated with higher risk for LBW [19]. The effect of maternal age on LBW infants was strong in both ends of the age spectrum; younger and older pregnant women tend to have higher risk for LBW. Nutritional depletion that is usually present among young pregnant women may be a one of the contributing factor [19]. On the other hand, advancing maternal age was associated with a decreased potential for fetal growth, possibly reflecting biological aging of maternal tissues and systems or the cumulative effects of disease [34]. It was shown in an earlier study that the effect of maternal age was highly significant for pregnant women who reside in low-income rural areas compared to high-income urban areas [35]. This suggests that the effect of maternal age on LBW may depend on lifestyle factors associated with residential areas in context.

Our study adds to the body of knowledge of the positive association between parity and birth weight [18, 36]. It was previously shown that the association between parity and birth weight was non-linear with the greatest increase observed between first and second birth [36]. A case-control study in Malaysia involving 350 women with LBW infants and 350 women as controls demonstrated higher odds for LBW in nulliparous women [18]. In the effect of parity on LBW, each subsequent pregnancy may have improved maternal body efficiency. The uteroplacental blood flow, responsible for delivering oxygen and nutrients to the fetus, is more efficient during subsequent pregnancies [37].

MUAC is an indicator of maternal fat, lean tissue stores, protein and energy reserves in the body [38]. Earlier studies have shown that MUAC was associated with LBW and intra-uterine growth restriction [39]. Pregnant women with low MUAC may have less protein reserves, which affect the intrauterine growth of the fetus [40]. Since a low MUAC measurement reflects low lean muscle mass and/or loss of subcutaneous fat [41], it is possible that high MUAC indicates adequate protein availability for fetal growth, which may explain the lower risk of LBW for women with high MUAC shown in the current study. The etiology of LBW is multifactorial and these factors are often interrelated [42]. It was demonstrated that the trend of maternal aging on the increased risk of LBW was highly significant for first birth but not second births [43]. Additionally, a study conducted in Japan showed a combined additive effect of pre-pregnancy BMI and parity on LBW: underweight women on their first pregnancy had an increased risk for LBW [44].

However, we did not include pre-pregnancy BMI and gestational weight gain in the logistic model as they were not correlated with birth weight in univariate analysis. There is an increasing evidence that upper body adiposity is operationalized for fetal growth as opposed to lower body adiposity that primarily contributes to lactation and has little or no effect on infant’s birth weight [45, 46]. MUAC is a measure of regional adiposity whereas BMI is a measure of overall adiposity [47]. Thus, the effect of BMI on birth weight may be diluted, as observed in the lack of correlation between BMI and birth weight in current study. It was shown earlier that the discrepancy of maternal upper- and lower-body fat led to disparities in infant’s birth weight between women of similar pre-pregnancy BMI and GWG [48]. Besides, it should be highlighted that MUAC is a measure of nutritional status before and during pregnancy, whereas pre-pregnancy BMI is a measure of nutritional status prior to pregnancy and gestational weight gain a measure during pregnancy. The nutritional status of women in the periconceptional period and throughout pregnancy is important for maternal and infant health. Our findings suggest that the emphasis should be on the nutritional status of rural pregnant women before and during pregnancy to reduce risk of LBW infants.

This current study has several limitations. First, the study was only conducted in one state of Malaysia, thus results may not be generalizable to all pregnant women in Malaysia. The loss to follow up due to high dropout rate may have introduced selection bias. In addition, physical activity and pre-pregnancy weight were self-reported and subjected to recall and social desirability bias. Objective measures should be included in future studies to accurately capture physical activity in addition to using the PPAQ. We recommend including other factors that may lead to LBW such as maternal diet, lifestyle factors (alcohol, tobacco, drug use) and complications during pregnancy such as hypertension [1] that were not part of our study. Strengths of this study include the use of a validated physical activity questionnaire designed specifically for pregnant women with consideration of type and intensity of physical activity. We also used two measures of nutritional status among pregnant women, pre-pregnancy BMI and MUAC. Lastly, this is among the first studies comparing maternal physical activity and risk factors for LBW of urban and rural pregnant women in Malaysia.

## Conclusions

In this study, we identified low MUAC, low parity, and greater maternal age as risk factors for LBW in rural, but not urban, pregnant women in Malaysia. Maternal physical activity was not associated with LBW in rural or urban women. Our findings provide pregnant women and health professionals in the middle-income countries with important information, which can inform maternal healthcare policy and interventions to reduce the risk of LBW infants by monitoring nutritional status during periconceptional period and throughout pregnancy especially for high-risk individuals such as rural pregnant women with low parity and older maternal age. Strategies to promote optimal nutritional status among malnourished women are needed before and during pregnancy, particularly in rural communities. Rural pregnant women with poor nutritional status should be encouraged to obtain sufficient protein and energy intake, and to monitor their MUAC consistently during pregnancy for reduced risk of LBW infants. This study is aligned with the National Plan of Action for Nutrition of Malaysia to promote maternal and infant nutrition to reduce risk of LBW infants to not more than 8% by 2025 by identifying and ensuring optimal care for high risk individuals [49]. Policymakers should be aware of the need for quality healthcare, nutrition education and lifestyle changes especially for women at high-risk for LBW.

## Abbreviations

BMI: Body Mass Index
LBW: Low birth weight
LMP: Last menstrual period
MET: Metabolic equivalent
MREC: Medical Research Ethics Committee
MUAC: Middle-upper arm circumference
NHMS: National and Health Morbidity Survey
NMRR: National Medical Research Registrar
PPAQ: Pregnancy Physical Activity Questionnaire
RM: Ringgit Malaysia
WHO: World Health Organization
